# Giant coronary aneurysm in IgG4–related disease

**DOI:** 10.1093/ehjcr/ytae131

**Published:** 2024-03-14

**Authors:** Yoann Roubertou, Romain Euvrard

**Affiliations:** Department of Internal and Vascular Medicine, Centre Hospitalier Lyon Sud, Hospices Civils de Lyon, 165 chemin du Grand Revoyet, Pierre-Bénite 69310, France; Department of Internal and Vascular Medicine, Centre Hospitalier Lyon Sud, Hospices Civils de Lyon, 165 chemin du Grand Revoyet, Pierre-Bénite 69310, France

A 62-year-old man was referred to the hospital for recurrent pancreatitis. He had a medical history of hypertension, diabetes treated with insulin, and excessive alcohol consumption. A computed tomography (CT) scan was performed, showing mediastinal, hilar, and retroperitoneal lymphadenopathy. Pancreatic magnetic resonance imaging found features of autoimmune pancreatitis and a cirrhotic liver. A positron emission tomography (PET) scan found uptake in the thoracic lymph nodes (asterisk, *Figure 1A*), the anterior wall of the left ventricle, and a saccular aneurysm of 16 mm in diameter located on the infra-renal abdominal aorta (arrow, *Figure 1A*). The initial electrocardiogram showed sinus rhythm without ischaemic sign. A coronary angiography was performed, revealing a giant aneurysm, 20 mm in diameter, on the left anterior descending artery (arrow, *Figure 1B*). Laboratory tests revealed elevated IgG4 (8587 mg/L; normal value < 864 mg/L). Syphilis and intracellular bacteria serologies, including Coxiella and Bartonella, were negative. The blood culture was sterile. There was no clinical or biological evidence for other systemic vasculitis, such as antineutrophil cytoplasmic antibodies (ANCA)-associated vasculitis, giant cell arteritis, or Behcet disease. An IgG4-related disease with pancreatic, lymphatic, aortic, and coronary involvement was diagnosed. The patient received corticosteroids with a total regression of the uptake on the PET scan performed at 2 months. The patient did not receive any endovascular or surgical treatment of the aneurysm because of the major bleeding risk linked to cirrhosis and thrombocytopenia.

**Figure 1:**
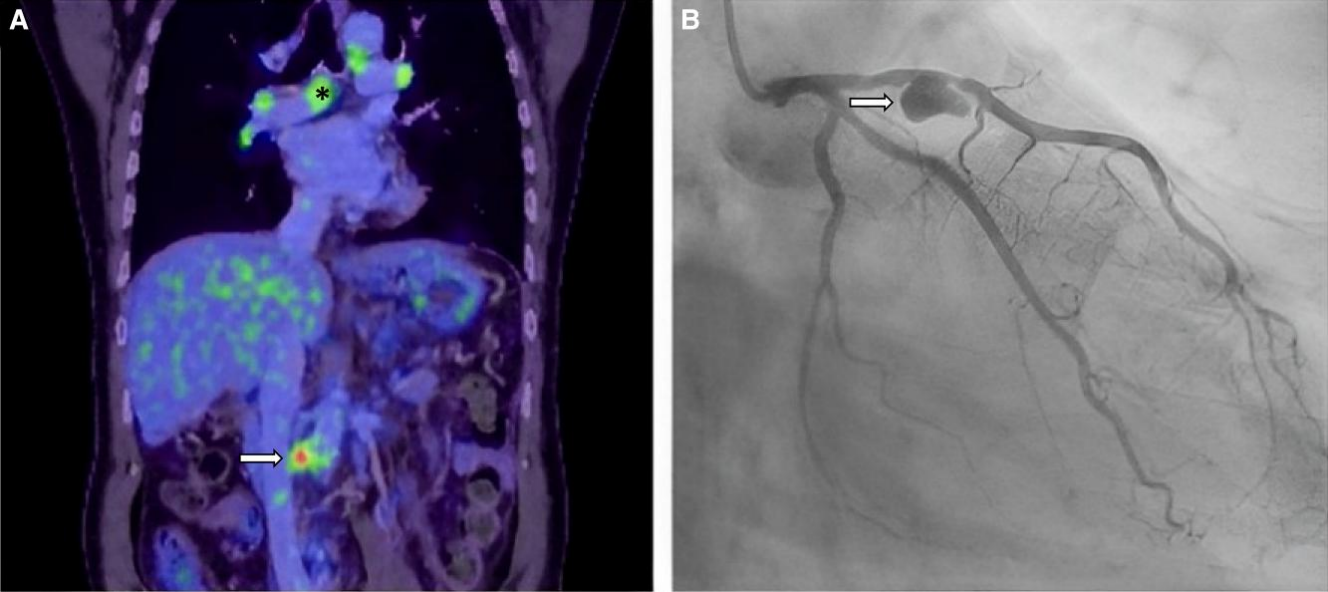
Giant coronary aneurysm in immunoglobulin G4–related disease. (*A*) Positron emission tomography scan showing aortic aneurysm (white arrow) and hilar and mediastinal lymphadenopathy (asterisk). (*B*) Coronarography showing a giant aneurysm (white arrow).

Immunoglobulin G4–related disease is a multi-organ condition which can lead to fibro-inflammatory lesions in almost every organ.^1^ The aetiology of the disease is unclear but it is presumed to be immune mediated. The variable presentation of the disease may be confused with other inflammatory diseases, such as sarcoidosis or vasculitis, infectious disease, or lymphoma. We report herein a rare vascular presentation of IgG4-related disease. Ito *et al.*^2^ described the largest case series of 42 patients with coronary aneurysm and IgG4-related disease. Most patients were asymptomatic (22/42, 52%), and coronary aneurysms were associated with peri-aortitis in half of the cases. Only three deaths were reported.

Coronary aneurysm in IgG4-related disease is a scarce and probably underdiagnosed condition. Physicians should be aware of this complication, especially in cases of IgG4-related peri-aortitis. A coronary CT could be proposed in IgG4-related peri-aortitis in order to allow the early detection of coronary aneurysms.

**Consent:** The authors confirm that written consent for the submission and publication of this case report, including images and associated text, was obtained from the patient.

**Funding:** None declared.

## Data Availability

More data underlying this article are available upon request with the corresponding author.
